# N-Acetylcysteine Alleviates Necrotizing Enterocolitis by Depressing SESN2 Expression to Inhibit Ferroptosis in Intestinal Epithelial Cells

**DOI:** 10.1007/s10753-024-02068-5

**Published:** 2024-07-22

**Authors:** Chuchu Gao, Lixia Wang, Kai Fu, Shan Cheng, Sannan Wang, Zongtai Feng, Shenglin Yu, Zuming Yang

**Affiliations:** 1 Department of Neonatology, The Affiliated Suzhou Hospital of Nanjing Medical University (Suzhou Municipal Hospital), Suzhou, 215002 China; 2 Department of Neonatology, Children’s Hospital of Soochow University, Suzhou, 215025 China; 3 Department of Urology, The Second Affiliated Hospital of Soochow University, Suzhou, 215004 China; 4 Department of Obstetrics and Gynecology, The Second Affiliated Hospital of Soochow University, Suzhou, 215004 China; 5 Department of Neurosurgery, The Second Affiliated Hospital of Soochow University, Suzhou, 215004 China

**Keywords:** Necrotizing enterocolitis, N-acetylcysteine, Ferroptosis, Sestrin2

## Abstract

*Abstract*-Necrotizing enterocolitis (NEC) is a severe gastrointestinal disease in neonates, and effective strategies to prevent and treat NEC are still lacking. Studies have shown that N-acetylcysteine (NAC) has protective effects against NEC, however, the specific mechanism underlying its effects on intestinal functions remains unclear. Recently, NAC has been shown to suppress ferroptosis in many diseases, while it is unclear whether the beneficial effects of NAC on NEC are related to ferroptosis. In this study, we revealed that ferroptosis was significantly induced in intestinal samples from infants with NEC. NAC alleviated intestinal inflammation, barrier damage and ferroptosis in multifactorial NEC models *in vivo* and *in vitro*. Sestrin2 (SESN2) was identified as an important mediator of NAC-induced ferroptosis resistance in intestinal epithelial cells. Furthermore, SESN2 knockdown inhibited the inflammatory response, alleviated barrier damage and ferroptosis in intestinal epithelial cells and enhanced the protective effects of NAC to a certain extent. Conversely, cells overexpressing SESN2 showed the opposite changes. In summary, our study demonstrated that NAC attenuates NEC progression by decreasing SESN2 expression to inhibit ferroptosis in intestinal epithelial cells, suggesting that NAC might be an effective clinical treatment for NEC.

**Supplementary Information:**

The online version contains supplementary material available at 10.1007/s10753-024-02068-5.

## INTRODUCTION

Necrotizing enterocolitis (NEC) is a severe intestinal disease that predominantly affects premature infants, resulting in high mortality rates and long-term complications [1, 2]. Infants diagnosed with NEC often must undergo aggressive medical interventions such as fasting, antibiotics or surgery [3]. Furthermore, some survivors may experience lasting issues such as narrow intestines, shortened bowels and developmental disorders, which seriously affect the quality of life of preterm infants [4, 5]. Despite rapid advancements in newborn care, there is still much to know about the pathogenesis of NEC, and finding ways to prevent and treat it remains a global challenge [4, 6]. The process of cell death is the key to understanding acute and chronic damage to the intestines [7]. Previous studies revealed that cells undergo multiple death pathways during NEC, mainly involving apoptosis and necrosis [8–10]. An imbalance in cell death pathways affects intestinal permeability and barrier function, ultimately leading to the occurrence of NEC [8, 11]. Therefore, in-depth research on the mechanisms underlying intestinal epithelial cell death holds promise for the development of innovative therapeutic approaches to combat NEC.

Ferroptosis is a newly discovered type of iron-dependent, caspase-dependent and lipid peroxidation-mediated programmed cell death [12]. In contrast to other cell death modes such as apoptosis and necrosis, ferroptosis is characterized by a shrinking of mitochondria and an increase in mitochondrial membrane density [13, 14]. The biochemical processes associated with ferroptosis mainly include the formation of iron-catalyzed lipid free radicals paired with the consumption of glutathione (GSH) or deactivation of the lipid repair enzyme GSH peroxygenase 4 (GPX4), which ultimately destroys the intestinal mucosal barrier [15]. In recent years, many studies have shown that ferroptosis plays a role in intestinal ischemia‒reperfusion injury and that inhibiting ferroptosis can alleviate intestinal injury [16, 17], offering a fresh viewpoint for managing acute and chronic intestinal disorders. Nonetheless, there is a scarcity of evidence suggesting that ferroptosis occurs in NEC, and the specific mechanism involved remains poorly understood.

Sestrin2 (SESN2, also called Hi95) is a highly conserved stress response protein that can be induced under various stress conditions, including hypoxia, DNA damage and oxidative stress [18, 19]. Numerous studies have demonstrated that SESN2 mediates endoplasmic reticulum stress, apoptosis and autophagy, hence serving as a major defense mechanism for the body against various stimuli [20]. However, there are few reports on the role of SESN2 in regulating ferroptosis, and further exploration is needed. The roles of SESN2 in liver, immune system and nervous system diseases have been thoroughly investigated, but little is known about its participation in intestinal system diseases.

N-acetylcysteine (NAC) is an antioxidative agent that has been shown to be effective in treating various pulmonary, gastrointestinal, neurological, psychiatric and metabolic diseases [21]. Although earlier studies have demonstrated the potential of NAC to mitigate intestinal damage severity and reduce mortality in experimental NEC [22, 23], the specific mechanisms underlying the beneficial effects of NAC are not yet clear. Recently, NAC was demonstrated to suppress ferroptosis in the uterus and placenta of rats with polycystic ovary syndrome [24]. NAC has also been proven to alleviate ferroptosis in diabetic nephropathy through the activation of the SIRT3-SOD2/GPX4 pathway to maintain mitochondrial redox [25]. Therefore, we speculated that the protective effect of NAC against NEC is related to ferroptosis, but further exploration is required to elucidate the mechanism underlying this effect.

In this work, we demonstrated that ferroptosis is involved in the pathophysiology of NEC. Furthermore, we evaluated whether NAC alleviates NEC by decreasing SESN2 expression to inhibit ferroptosis. Our study deepens the understanding of the role of ferroptosis in NEC and evaluates the underlying mechanism by which NAC alleviates NEC.

## MATERIALS AND METHODS

### Reagents and Antibodies

Lipopolysaccharide (LPS), cobalt chloride (CoCl_2_), and deferoxamine (DFO) were procured from Sigma‒Aldrich (St. Louis, MO, USA). NAC and ferrostatin-1 (Fer-1) were obtained from Selleckchem (Houston, TX, USA). Antibodies against GPX4 (ab125066), Tfrc (ab269513), PTGS2 (ab62331), FTH1 (ab183781), FTL (ab109373), SLC7A11 (ab175186), SESN2 (ab178518) and 4-HNE (ab46545) were procured from Abcam (Cambridge, UK). E-cadherin antibody (#14,472) was sourced from Cell Signaling Technology (Danvers, MA, USA), while ZO-1 (21,773–1-AP) and Claudin-1 (13,050–1-AP) antibodies were acquired from Proteintech (Wuhan, China). Furthermore, *β*-actin antibody (AC026) was obtained from ABclonal Technology (Wuhan, China). HRP-conjugated secondary antibodies (GAM007 and GAR007) were procured from MultiSciences (Hangzhou, China).

### Human Tissue Samples

Specimens from infants with NEC (*n* = 11) and from “noninflammatory” control infants (*n* = 13), including those with intestinal perforation (*n* = 1), volvulus (*n* = 1), congenital small bowel atresia (*n* = 2), intestinal obstruction (*n* = 2) and intestinal malrotation (*n* = 7), were obtained from the Department of General Surgery at Children’s Hospital of Soochow University. The use of these tissue samples was sanctioned by the Medical Ethics Committee of the Children’s Hospital of Soochow University (CS180), with all guardians providing written informed consent. This study was carried out in adherence to the principles of the Declaration of Helsinki. The baseline characteristics of the infants with NEC and control infants are detailed in Table 1.

**Table 1:** Characteristics of NEC and Control Infants

	NEC (*n* = 11)	Control (*n* = 13)
Sex, %(n)		
Male	72.7(8)	76.9(10)
Female	27.3(3)	23.1(3)
Gestational age (weeks), median (IQR^a^)	30.7(28.4–31.9)	39.0(37.7–39.9)
Delivery mode, %(n)		
vaginal	63.6(7)	38.5(5)
C-section	36.4(4)	61.5(8)
Apgar score (≤ 7) at 1 min, %(n)	18.2(2)	7.7(1)
Amniotic fluid-pollution, %(n)	0(0)	15.4(2)
Birth weight (g), median (IQR)	1240(920–1400)	3550(2830–3865)
SGA^a^, %(n)	9.1(1)	0(0)
Pregnancy status, %(n)		
Multiple pregnancy	27.3(3)	7.7(1)
GDM^a^	9.1(1)	15.4(2)
GH^a^	27.3(3)	7.7(1)
PROM^a^	18.2(2)	7.7(1)
With PDA^a^, %(n)	54.5(6)	15.4(2)
Previously infused with RBC^a^ suspension, %(n)	72.7(8)	0(0)
Feeding breast milk, %(n)	9.1(1)	NA^a^
Postnatal age of onset (d), median (IQR)	27(10–37)	2(0–9)
Laboratory features, median (IQR)		
WBC^a^ (× 10^9^/L)	5.4(3.1–14.7)	10.98(7.2–15.8)
PLT^a^ (× 10^9^/L)	112(80–272)	297(210–345)
hs-CRP^a^ (mg/L)	56.7(25.2–169.7)	7.1(0.8–19.3)

^*^IQR Interquartile range, *SGA* Small for gestational age, *GDM* Gestational diabetes mellitus, *GH* Gestational hypertension, *PROM* Premature rupture of membranes, *PDA* Patent ductus arteriosus, *RBC* Red blood cell, *NA* Not applicable, *WBC* White blood cells, *PLT* Platelet, *hs-CRP* Hypersensitive C-reactive protein

### Animal Experiments

The animal experiments were reviewed and approved by the Ethics Committee of Soochow University (SUDA20230719A01). C57BL/6 mouse pups (five to seven days old) were purchased from JOINN Laboratories (Suzhou, China), and experimental NEC was induced as per established protocols [26]. Briefly, the mice were fed a specific formula (a combination of 10 g of Similac Plus [Abbott, Saint-Laurent, Canada] and 50 ml of Esbilac [PetAg, Hampshire, USA]), 40 μl/g through gavage every 6 h, followed by exposure to hypoxia (95% N_2_ + 5% O_2_ for 10 min after half an hour of feeding) and intragastric administration of LPS (4 μg/g added to the second feeding of formula on the second and third days of NEC modeling). The pups were randomly assigned to four experimental groups: the control group (Group 1), which included pups that were breastfed by their mothers; the NAC group (Group 2), which included pups that stayed with their mothers and were administered NAC (150 μg/g of body weight) by intraperitoneal injection every day; the NEC group (Group 3), which included pups that were separated from their mothers and used to model NEC by the method described above; and the NAC + NEC group (Group 4), which included pups that were separated from their mothers and subjected to the same conditions as Groups 2 and 3. The surviving pups in all groups were euthanized after 96 h to collect ileal tissues and serum samples.

### Cell Culture and Treatments

FuHeng Biotechnology Co., Ltd., provided the IEC-6 cell line (RRID: CVCL_0343), which was cultured in high-glucose DMEM (HyClone, UT, USA) supplemented with 10% fetal bovine serum (Gibco, USA) and maintained in an incubator at 37 °C with 5% CO_2_. The cells were exposed to LPS (10 μg/mL) and CoCl_2_ (400 μM) for 24 h to establish the *in vitro* NEC model. Unless specified otherwise, the cells were subjected to 2 μM Fer-1, 200 μM DFO or 3 mM NAC for 2 h before LPS stimulation.

### Cell Transfection

Control small interfering RNA (siRNA) and SESN2 siRNAs were obtained from GenePharma (Shanghai, China). The sequences can be found in Table 2. SESN2 cDNA was cloned and inserted into a pcDNA3.1 expression vector expressing a Flag tag for transient transfection to establish SESN2-overexpressing cells. The empty vector and SESN2 plasmid were purchased from Youbio Biological Technology (Hunan, China). Transient transfection was conducted utilizing Lipofectamine™ 3000 (Invitrogen, USA), and the transfected cells were used in the subsequent experiments.

**Table 2:** SESN2 mRNA-Specific siRNAs and Negative Control (NC) siRNA

	Sense(5' to 3')	Antisense(5' to 3')
si-SESN2-1	CCCAGACAUCCUAUGCUUUTT	AAAGCAUAGGAUGUCUGGGTT
si-SESN2-2	GGAACCUCAAGAUCUAUAUTT	AUAUAGAUCUUGAGGUUCCTT
si-SESN2-3	GCAAGCAGCUUUGCUCUAUTT	AUAGAGCAAAGCUGCUUGCTT
NC	UUCUCCGAACGUGUCACGUTT	ACGUGACACGUUCGGAGAATT

### Cell Viability

IEC-6 cells were seeded in 96-well plates at a density of 8,000 cells per well (BD Falcon, Corning Inc., Corning, NY) and cultured to form a monolayer for approximately 48 h. The medium was then changed to serum-free medium. After treatment, cell viability was determined utilizing a cell counting kit-8 (CCK-8, NCM Biotech, Suzhou, China).

### Hematoxylin and Eosin (H&E) Staining

The collected ileum samples were fixed in 4% paraformaldehyde, embedded in paraffin blocks, sectioned at 5 μm, and stained with H&E. Based on the published scoring system, intestinal injury severity scores were assessed in a blinded manner by histological evaluation.

### Immunohistochemistry (IHC)

Intestinal tissue was fixed in 4% paraformaldehyde, embedded in paraffin, dewaxed, and subjected to antigen retrieval. To inhibit endogenous peroxidase activity, 3% H_2_O_2_ and 5% BSA were used successively. Subsequently, primary antibody incubation was performed. Visualization was performed using an immunochromogenic kit (Gene Tech, Shanghai, China). A secondary antibody was applied, followed by the addition of DAB color development solution. After a few minutes, the slices were washed and stained with hematoxylin. After washing, the slices were dehydrated using graded ethanol solutions, followed by clearing with xylene and sealing with neutral resin. Finally, images of these slices were taken under a light microscope.

### Immunofluorescence (IF)

Intestinal tissues that had undergone antigen repair and fixed IEC-6 cells were blocked with 5% BSA before being incubated with the indicated primary antibodies. After washing with PBS, the specimens were incubated in the dark with a secondary antibody labeled with fluorescent dye. Nuclei were stained with DAPI for 5 min. Fluorescence images were acquired using a Zeiss confocal microscope (Zeiss Microsystems, Germany).

### Enzyme-linked Immunosorbent Assay (ELISA)

ELISA kits (RK00020, ABclonal, Wuhan, China; P16599, CUSABIO, Wuhan, China) were used to assess the amounts of IL-6 and TNFα in the cell culture supernatant.

### Quantitative Reverse Transcription-polymerase Chain Reaction (qRT‒PCR)

TRIzol reagent (Invitrogen, Carlsbad, CA, USA) was utilized to extract the total RNA, and a cDNA 1st Strand Synthesis kit (Novoprotein, China) was used to reverse transcribe the RNA into cDNA. SYBR qPCR SuperMix (Novoprotein, China) was used for qRT‒PCR. Utilizing the 2^−△△Ct^ technique, mRNA quantities were assessed and adjusted against *β*‐actin levels for normalization. The sequences for both the forward (F) and reverse (R) primers are provided in Table 3.

**Table 3:** Primers Used in This Study

Gene	Forward(5' to 3')	Reverse(5' to 3')
IL-6	CCGGAGAGGAGACTTCACAG	ACAGTGCATCATCGCTGTTC
TNFα	GAAAAGCAAGCAACCAGCCA	CGGATCATGCTTTCCGTGCTC
Claudin-1	GGATGGATCGGCTCTATCGTCA	GATGGCCTGAGCAGTCACGAT
ZO-1	GACCCTGACCCAGTGTCTGATAA	CTATCCCTTGCCCAGCTCTTCT
SESN2	TCAGCGGATATTCTGGAGCC	ATGGAGGTGTCTACGCCACT
*β*-actin	CACCCGCGAGTACAACCTTC	CCCATACCCACCATCACACC

### Western Blot Analysis

A protease inhibitor cocktail (ab271306, Abcam, Cambridge, UK) was added to RIPA buffer (Beyotime, Shanghai, China) for the extraction of total protein. Protein separation was achieved through SDS‒PAGE. The membranes were incubated with primary antibodies after 1 h in a blocking solution at room temperature. Subsequently, the membranes were incubated with an HRP-conjugated secondary antibody. Identification of the protein bands was performed using a Syngen GeneGnome XRQ system (Syngene, UK) and enhanced chemiluminescence (ECL) detection reagents (Yeasen Biotechnology, Shanghai, China).

### Wound Healing Assay

The cells were cultivated at a density of 5 × 10^5^ cells per well in 6-well plates (BD Falcon, Corning Inc., Corning, NY). Once reaching confluence, the cells were separated into groups and treated with different agents in serum-free medium following the application of a scratch through the layer of cells. The documentation of wound closure was carried out after 0 h and 24 h of incubation. The healed area of each wound was calculated using the following formula: acellular area at 24 h/acellular area at 0 h × 100%.

### RNA Sequencing (RNA-seq) ANALYSIS

RNA-seq was performed and the results were analyzed by LC Bio Technology (Hangzhou, China) on the Illumina NovaSeq™ 6000 system. For the differentially expressed genes (DEGs), both Gene Ontology (GO) enrichment and Kyoto Encyclopedia of Genes and Genomes (KEGG) pathway analyses were conducted.

### Transmission Electron Microscopy Imaging

After fixation with electron microscopy fixative, IEC-6 cells were collected and centrifuged. Subsequently, the samples were postfixed with 1% osmium tetroxide, dehydrated with ethanol and acetone and embedded in epoxy resin. Polymerization was carried out at 60 ℃ for 48 h, followed by the cutting of ultrathin Sects. (60 nm thick) and staining with 2% uranyl acetate and lead citrate. The cellular ultrastructural morphology was observed by TEM, and images were taken.

### Measurement of the Reduced Glutathione (GSH)/Oxidized Glutathione Disulfide (GSSG) Ratio

The GSH/GSSG ratio was evaluated using a GSH and GSSG assay kit (Beyotime, Shanghai, China) according to the manufacturer’s protocols. The concentrations of total glutathione and GSSG were measured successively at 412 nm by using an enzyme labeling instrument. The GSH/GSSG ratio was calculated using the following formula: (total glutathione—GSSG × 2)/GSSG.

### MDA Measurement

The relative MDA concentration was measured with a lipid peroxidation MDA assay kit (Beyotime, Shanghai, China). Tissues or cells were homogenized or lysed before centrifugation to obtain the supernatant. Then, the supernatant was added to the MDA working solution and incubated at 100 ℃ for 15 min, after which the absorbance was measured at 532 nm.

### Lactate Dehydrogenase (LDH) Release Assay

IEC-6 cells grown in 96-well plates were treated and cultured for a period of time. Then, LDH release was determined utilizing a cytotoxicity LDH assay kit (Dojindo, Kumamoto, Japan). After the addition of lysis buffer or working solution to some wells, LDH levels in the medium were measured. Subsequently, the absorbance was measured at 490 nm.

### Assessment of Lipid Peroxidation by Flow Cytometry or Fluorescence Imaging

IEC-6 cells were incubated with Liperfluo (Dojindo, Kumamoto, Japan) at 37 °C for 0.5 h. Following incubation, the cells were rinsed, harvested, and resuspended in HHBS. A flow cytometer (Beckman, Germany) was used to quantify the cell fluorescence, which was analyzed using FlowJo software.

The cells were exposed to BODIPY™ 581/591 C11 (Thermo Fisher Scientific, MA, USA), followed by washing with PBS and staining of the nuclei. Fluorescence images were acquired by a Zeiss confocal microscope.

### Assessment of Intracellular Fe^2+^ by Fluorescence Imaging

IEC-6 cells were washed with HHBS and then treated with FerroOrange (Dojindo, Kumamoto, Japan) at 37 °C for 0.5 h. Cell fluorescence was visualized by a Zeiss confocal microscope.

### Molecular Docking

The stereo structures of the protein and compound were obtained from the UniProt and PubChem databases and then processed through the ADT tool. Subsequently, the parameters of the docking box were calculated by PyMol 2.2.0, which led to the docking and scoring executed by AutoDock Vina 1.2.3. Following the completion of docking, the results were examined and interpreted with PyMOL and Discovery Studio 2021.

### Statistical Analysis

Data analyses were carried out using GraphPad Prism 8.3.0. All of the data were obtained from at least three independent experiments. One-way ANOVA was used to compare normally distributed data between groups. The data in this study were expressed as the mean ± standard deviation. Using the nonparametric Mann‒Whitney *U* test, nonnormally distributed quantitative data were compared between different groups. These data were presented as the medians and interquartile ranges. The Kaplan‒Meier method was used to assess the survival data. A *p* value less than 0.05 was considered to indicate statistical significance.

## RESULTS

### Ferroptosis was Induced in Infants with NEC and in *in Vivo* and *in vitro* NEC Models

**Fig. 1:**
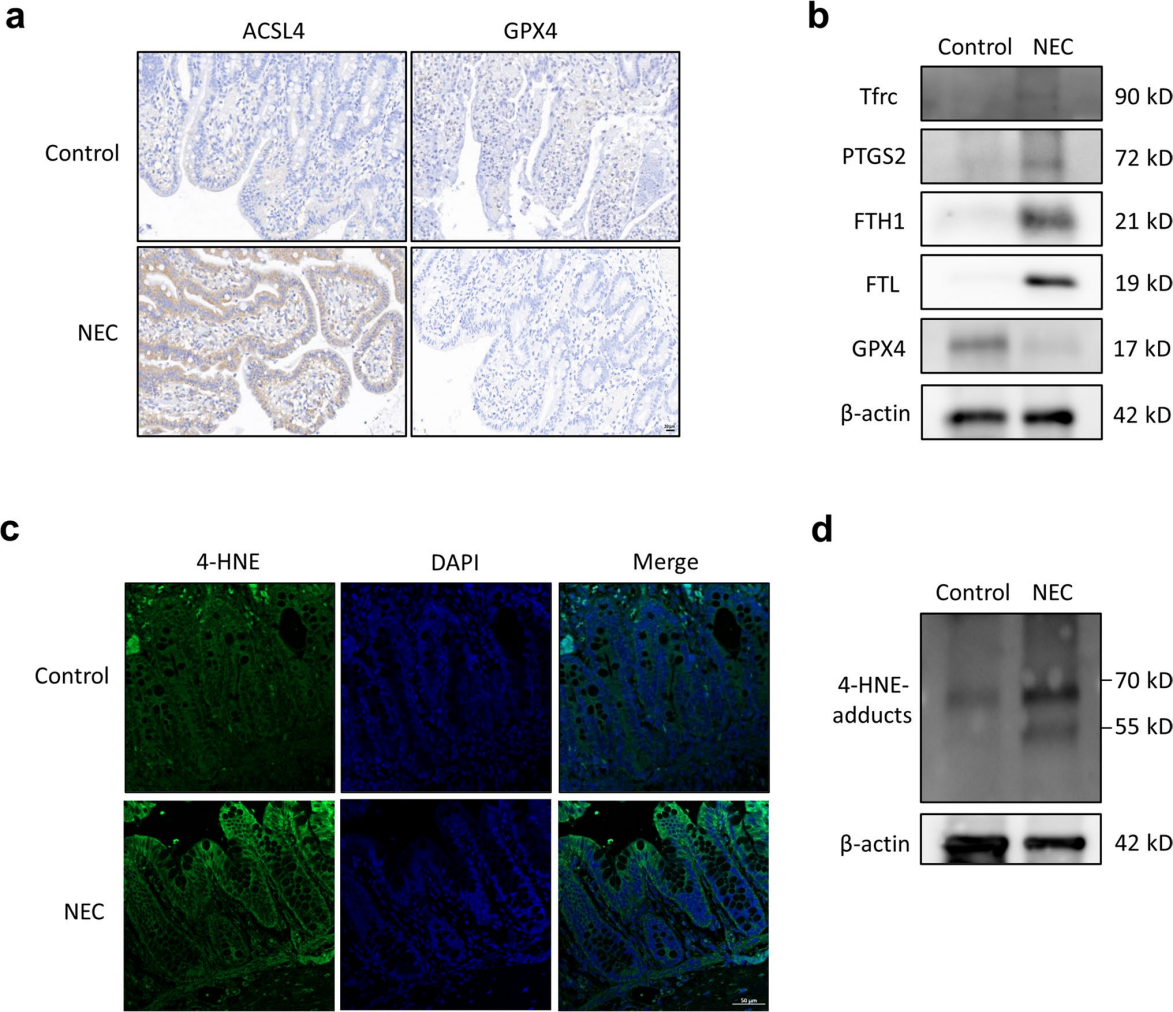
Ferroptosis participated in the pathogenesis of NEC. **a** Representative sections from IHC staining of intestinal tissues of control or NEC patients stained with anti-ACSL4 and anti-GPX4 antibodies. Scale bar = 20 µm. **b** Representative immunoblots of Tfrc, PTGS2, FTH1, FTL and GPX4 expression in intestinal tissues. **c** Representative IF images of 4-HNE staining in intestinal tissues. Scale bar = 50 µm. **d** Representative immunoblots of 4-HNE in intestinal tissues.

To determine whether ferroptosis is involved in the pathogenesis of NEC, we first assessed the levels of ferroptosis-associated proteins (ACSL4 and GPX4) in the intestinal tissues of infants by IHC. ACSL in the NEC group was increased compared with that in the control group, while GPX4 was decreased (Fig. 1a). We further examined the expression of several reported ferroptosis-associated proteins in the two groups. Western blotting confirmed that in the NEC group, Tfrc, PTGS2, FTH1, and FTL expression was elevated, while GPX4 expression was decreased (Fig. 1b). Moreover, we evaluated the expression of 4-HNE, which is a marker of lipid peroxidation. IF showed that 4-HNE staining was increased in the NEC group (Fig. 1c). Consistently, similar results were obtained via Western blot analysis of intestinal specimens (Fig. 1d). These findings revealed the participation of ferroptosis in the pathogenesis of NEC.

Based on the results observed in infants with NEC, we established an animal model using a previously reported method [26] to further confirm the induction of ferroptosis in NEC model mice. Mitochondrial morphological changes are considered a feature of ferroptosis. TEM revealed obvious changes in mitochondrial morphology, including decreased mitochondrial volume, increased membrane density, decreased number or lack of cristae, and outer mitochondrial membrane rupture, in terminal ileal tissue samples from NEC model mice (Fig. 5a). Since lipid peroxidation is a hallmark of ferroptosis, we measured the levels of 4-HNE and MDA, two lipid peroxidation products. IF and densitometry showed significant increases in 4-HNE and MDA in the intestinal tissue of NEC model mice (Fig. 5b-e). Moreover, we found that the levels of GSH and GSH/GSSG, important markers of lipid peroxidation, were obviously decreased in NEC model mice (Fig. 5c, d). In addition, we evaluated the protein levels of several core factors involved in regulating ferroptosis, including SLC7A11, FTH1, FTL and GPX4. These indicators were differentially expressed between NEC model mice and control mice (Fig. 5f, g). These data indicated that ferroptosis was induced in our *in vivo* model of NEC.

**Fig. 2:**
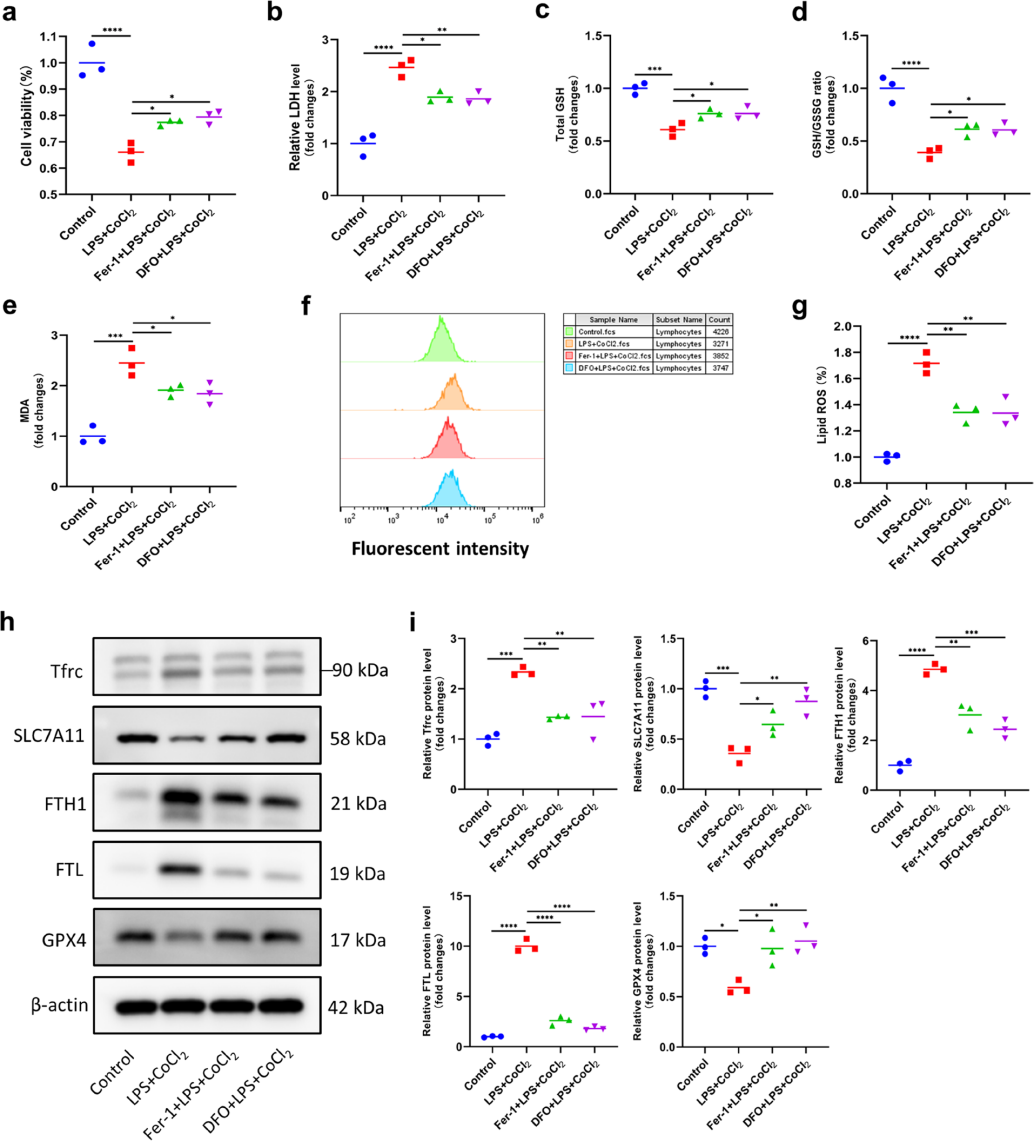
Ferroptosis existed in an *in vitro* intestinal injury model of NEC. **a** Cell viability of IEC-6 cells in the control, LPS + CoCl_2_, Fer-1 + LPS + CoCl_2_ and DFO + LPS + CoCl_2_ groups. **b** LDH levels of IEC-6 cells in different groups. **c-e** GSH levels, the GSH/GSSG ratio and MDA content in IEC-6 cells. **f, g** Lipid peroxidation levels of IEC-6 cells detected by flow cytometry. **h, i** Western blot analysis of Tfrc, SLC7A11, FTH1, FTL and GPX4 expression. ^*^p < 0.05, ^**^p < 0.01, ^***^p < 0.001, ^****^p < 0.0001.

Given that NEC is an intestinal disease caused by various factors, we established an *in vitro* NEC-related intestinal injury model by inducing inflammation with LPS and hypoxia with CoCl_2_ in IEC-6 cells. We used the ferroptosis inhibitors Fer-1 and DFO to verify the induction of ferroptosis in LPS + CoCl_2_-treated cells. As shown in Fig. 2, cell viability, GSH levels and the GSH/GSSG ratio in LPS + CoCl_2_-treated cells were significantly decreased (Fig. 2a, c, d), while LDH release and MDA and lipid peroxidation levels were significantly increased (Fig. 2b, e–g). Additionally, in IEC-6 cells treated with LPS + CoCl_2_, there was a significant decrease in the levels of the ferroptosis-related proteins SLC7A11 and GPX4 and a large increase in the levels of Tfrc, FTH1 and FTL (Fig. 2h, i). However, Fer-1 and DFO reversed all of these changes in IEC-6 cells (Fig. 2a-i). These results suggested that ferroptosis was induced in our in vitro NEC-related intestinal injury model.

Based on these results, we concluded that ferroptosis was induced in infants with NEC and *in vivo* and *in vitro* in NEC models.

### NAC Reduced the Severity of Experimental NEC *in Vivo* and *in Vitro*

**Fig. 3:**
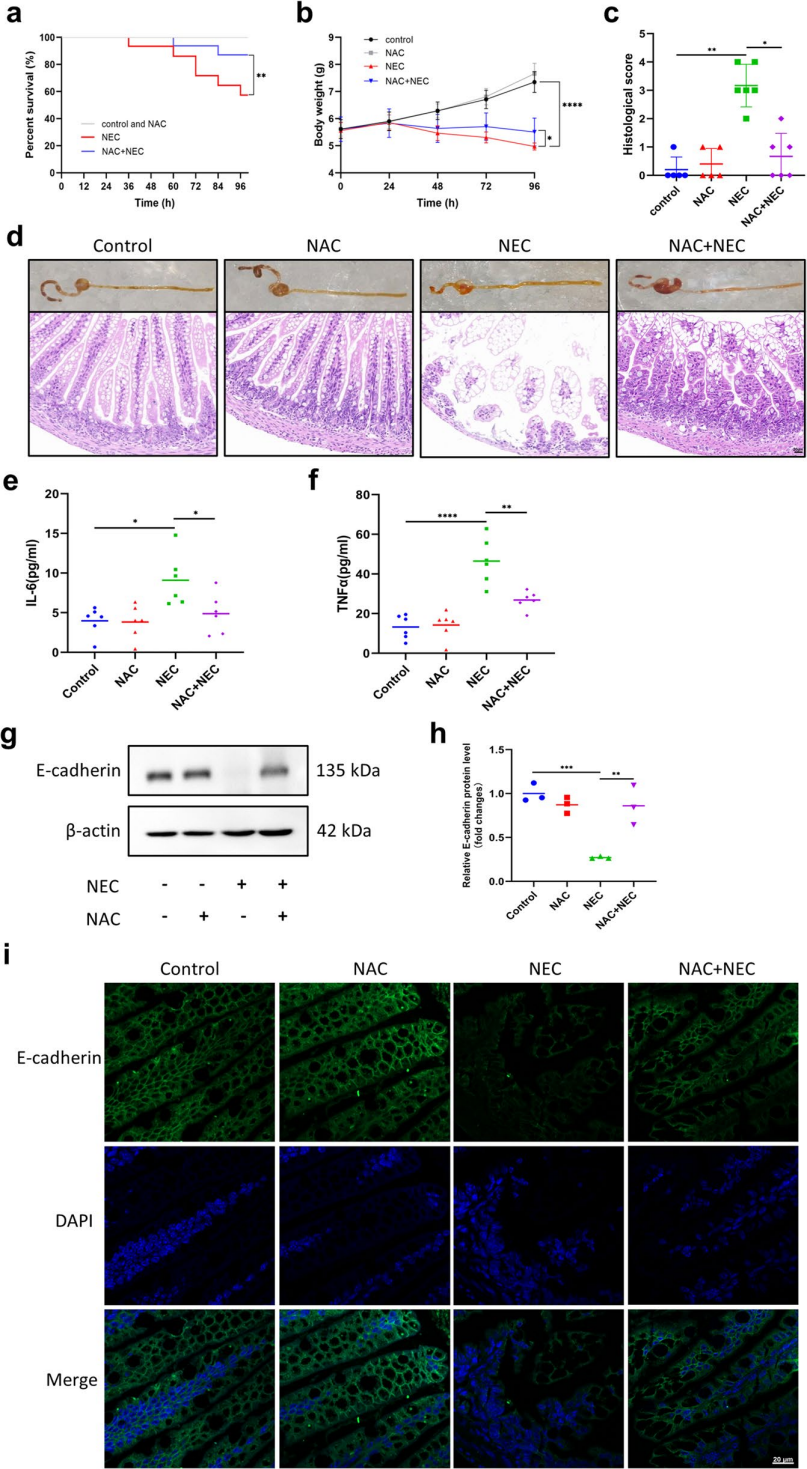
NAC reduced the severity of experimental NEC. **a** Kaplan‒Meier survival curves of mice in the control, NAC, NEC and NAC + NEC groups (*n* = 12 ~ 14 per group). **b** Body weight changes of mice in different groups (*n* = 8 ~ 9 per group). **c** Histological scores for different groups (*n* = 5 ~ 6 per group). **d** Representative images of the gross appearance and H&E staining of intestinal tissues. Scale bar = 20 µm. **e, f** Serum IL-6 and TNFα levels in different groups were detected by ELISA (*n* = 6 per group). **g, h** Western blot analysis of E-cadherin protein levels in intestinal tissues. **i** Representative IF images of E-cadherin staining in intestinal tissues. Scale bar = 20 µm. ^*^p < 0.05, ^**^p < 0.01, ^***^p < 0.001, ^****^p < 0.0001.

To determine whether NAC can confer protection against NEC *in vivo*, we used the experimental NEC mouse model mentioned above. The present study showed that NAC supplementation significantly increased the survival rate and attenuated weight loss in NEC model mice (Fig. 3a, b). Given that the extent of intestinal injury is an important criterion for evaluating NEC, we observed macroscopic changes in terminal ileal tissue samples and then used H&E staining to assess morphological alterations and histopathological scores to further quantify the degree of intestinal damage. According to the results, there were obvious gross lesions, edema, necrosis and shedding of intestinal villi in the NEC model mice, and the NEC group exhibited greater intestinal injury scores than did the control and NAC groups. However, the gross lesions of the mice in the NAC + NEC group were relatively mild, as the structure of the intestinal villi was intact, the degree of edema was significantly reduced, and histological injury was ameliorated (Fig. 3c, d). The above results indicated that NAC could reduce the severity of NEC.

Additionally, excessive inflammation and abnormal expression of intestinal barrier proteins are fundamental features of NEC. Subsequently, we assessed the accumulation of inflammatory cytokines and the expression of a barrier protein in the different groups. Our ELISA analysis demonstrated a noteworthy increase in the serum IL-6 and TNFα concentrations in the NEC group. Nevertheless, in the NAC + NEC group, these changes were obviously reversed (Fig. 3e, f). In addition, Western blotting and IF revealed a decrease in intestinal E-cadherin expression in the NEC group; however, NAC supplementation substantially reversed the suppression of E-cadherin expression in NEC model mice (Fig. 3g-i). These results suggested that NAC could reduce the severity of NEC by alleviating the inflammatory response and intestinal damage.

**Fig. 4:**
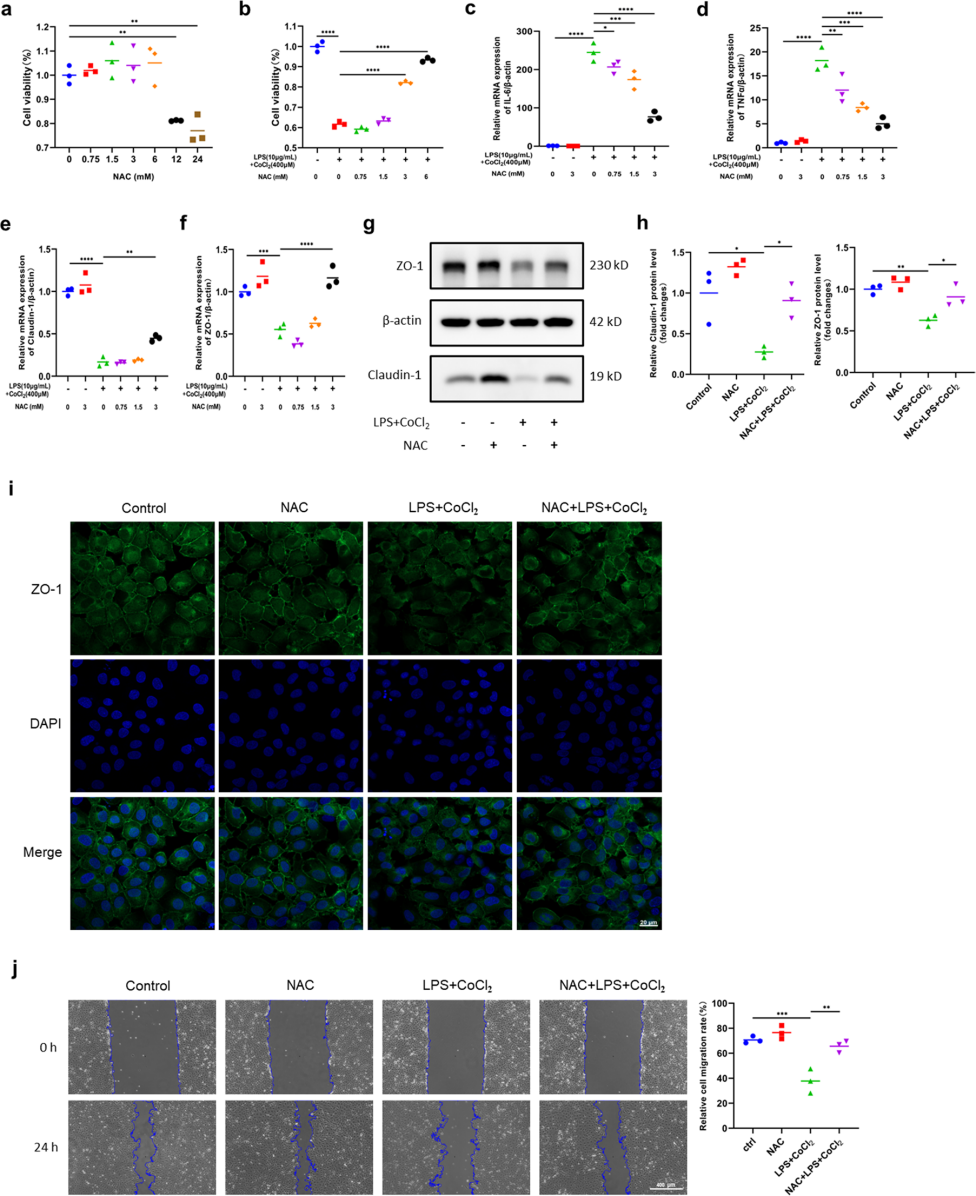
NAC alleviated the inflammatory response and barrier damage in IEC-6 cells. **a** Cell viability of IEC-6 cells treated with different concentrations of NAC. **b** Cell viability of IEC-6 cells treated with LPS + CoCl_2_ and pretreated with different concentrations of NAC. **c-f** mRNA levels of IL-6, TNFα, Claudin-1 and ZO-1 detected by qRT‒PCR. **g, h** Western blot analysis of Claudin-1 and ZO-1 expression. **i** Representative IF images of ZO-1 staining in IEC-6 cells. Scale bar = 20 µm. **j** Representative images of the cellular wound healing assay and quantitative analysis of the migration rates. Scale bar = 400 µm. ^*^p < 0.05, ^**^p < 0.01, ^***^p < 0.001, ^****^p < 0.0001.

To explore the protective effect of NAC against intestinal injury in vitro, we first determined that the safe concentration range of NAC in IEC-6 cells was 0–6 mM by using the CCK-8 assay (Fig. 4a). We then pretreated the cells with different concentrations of NAC in this range before inducing IEC-6 cell damage with LPS + CoCl_2_. The CCK-8 assay showed that NAC reversed the decrease in cell viability induced by LPS + CoCl_2_ in a dose-dependent manner (Fig. 4b). In addition, we evaluated the effect of different concentrations of NAC on the mRNA levels of inflammatory cytokines and barrier proteins. NAC markedly suppressed the release of IL-6 and TNFα induced by LPS + CoCl_2_ in a dose-dependent manner. Conversely, NAC reversed the reduction in the Claudin-1 and ZO-1 levels induced by LPS + CoCl_2_ to some extent (Fig. 4c-f). In addition, the above results indicated that 3 mM NAC maximally reduced IEC-6 cell inflammation and barrier damage. Furthermore, Western blotting and IF showed that NAC obviously alleviated the suppression of Claudin-1 and ZO-1 expression induced by LPS + CoCl_2_ (Fig. 4g-i). Moreover, the results of the wound healing assay confirmed that the wound closure ability of IEC-6 cells pretreated with NAC was improved (Fig. 4j). The above consequences suggested that NAC could alleviate the inflammatory response and barrier damage in intestinal cells in vitro.

### NAC Inhibited Ferroptosis in NEC Model Mice and IEC-6 Cells

**Fig. 5:**
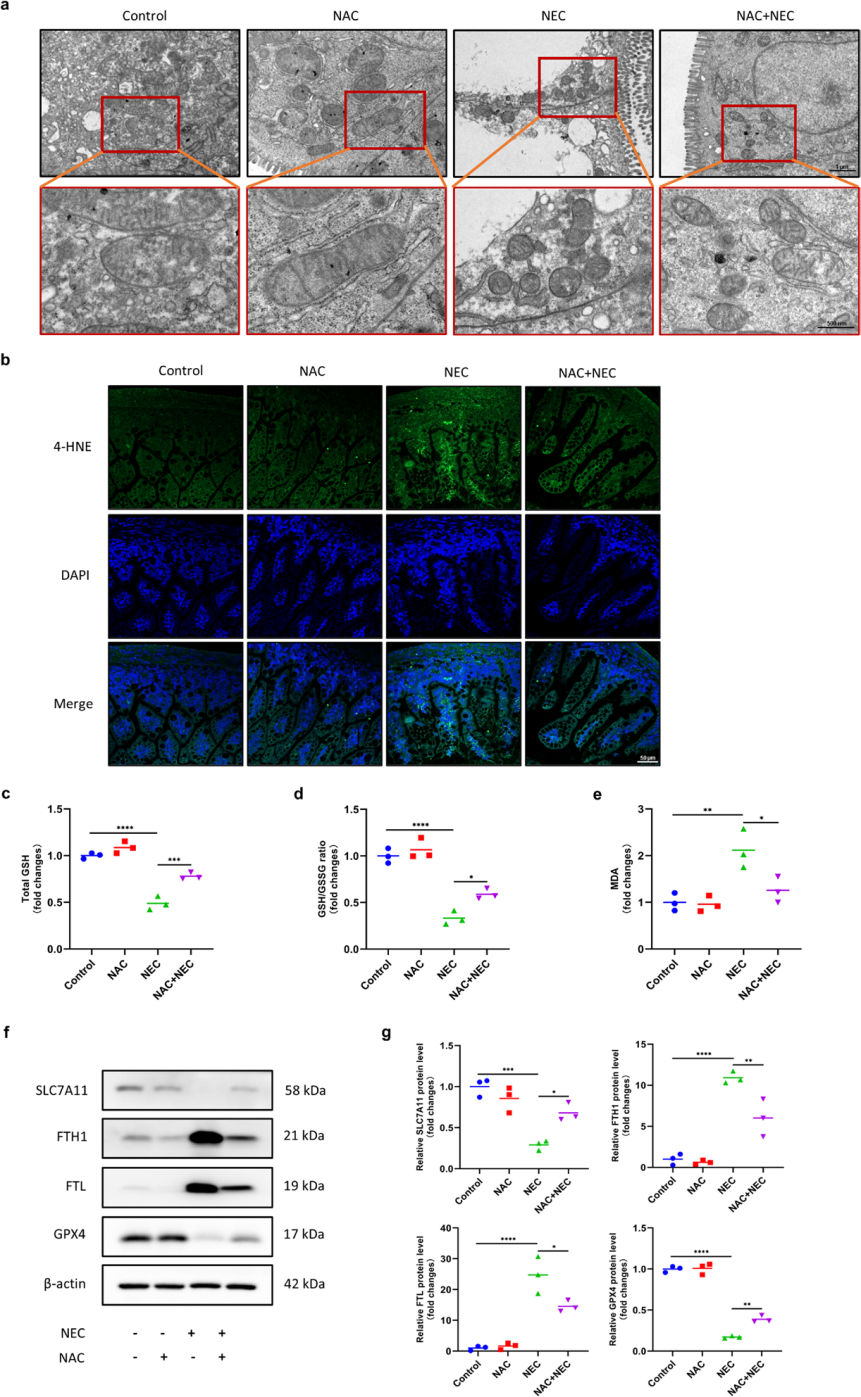
NAC inhibited ferroptosis in NEC mice. **a** Representative images of the ultrastructure of intestinal mitochondria by TEM. Scale bar = 1 µm (upper panel)/500 nm (lower panel, enlarged view). **b** Representative IF images of 4-HNE staining in intestinal tissues. **c-e** GSH levels, the GSH/GSSG ratio and the MDA content of intestinal tissues. **f, g** Western blot analysis of SLC7A11, FTH1, FTL and GPX4 expression in intestinal tissues. ^*^p < 0.05, ^**^p < 0.01, ^***^p < 0.001, ^****^p < 0.0001.

Using TEM to observe the mitochondrial morphology is the most direct method for detecting ferroptosis. The results showed that there were obvious changes in the mitochondrial morphology consistent with ferroptosis in the intestinal tissues of NEC model mice. In contrast, mitochondrial morphology in the NAC + NEC group showed milder changes, with a slight increase in the membrane density, clear cristae, and an intact outer membrane (Fig. 5a). Furthermore, we measured the levels of both 4-HNE and MDA. IF and densitometry showed that NAC significantly inhibited the increase in both 4-HNE and MDA levels in the intestinal tissue of NEC model mice (Fig. 5b, e). In addition, we measured GSH levels and the GSH/GSSG ratio and found that NAC inhibited the decrease in GSH levels and the GSH/GSSG ratio in NEC model mice, indicating its inhibitory effect on lipid peroxidation (Fig. 5c, d). Moreover, we evaluated the protein levels of SLC7A11, FTH1, FTL and GPX4. The results indicated that NAC effectively rescued SLC7A11 and GPX4 expression and suppressed FTH1 and FTL expression in NEC model mice (Fig. 5f, g). The findings of the *in vivo* study indicated that NAC could inhibit intestinal ferroptosis induced by NEC.

**Fig. 6:**
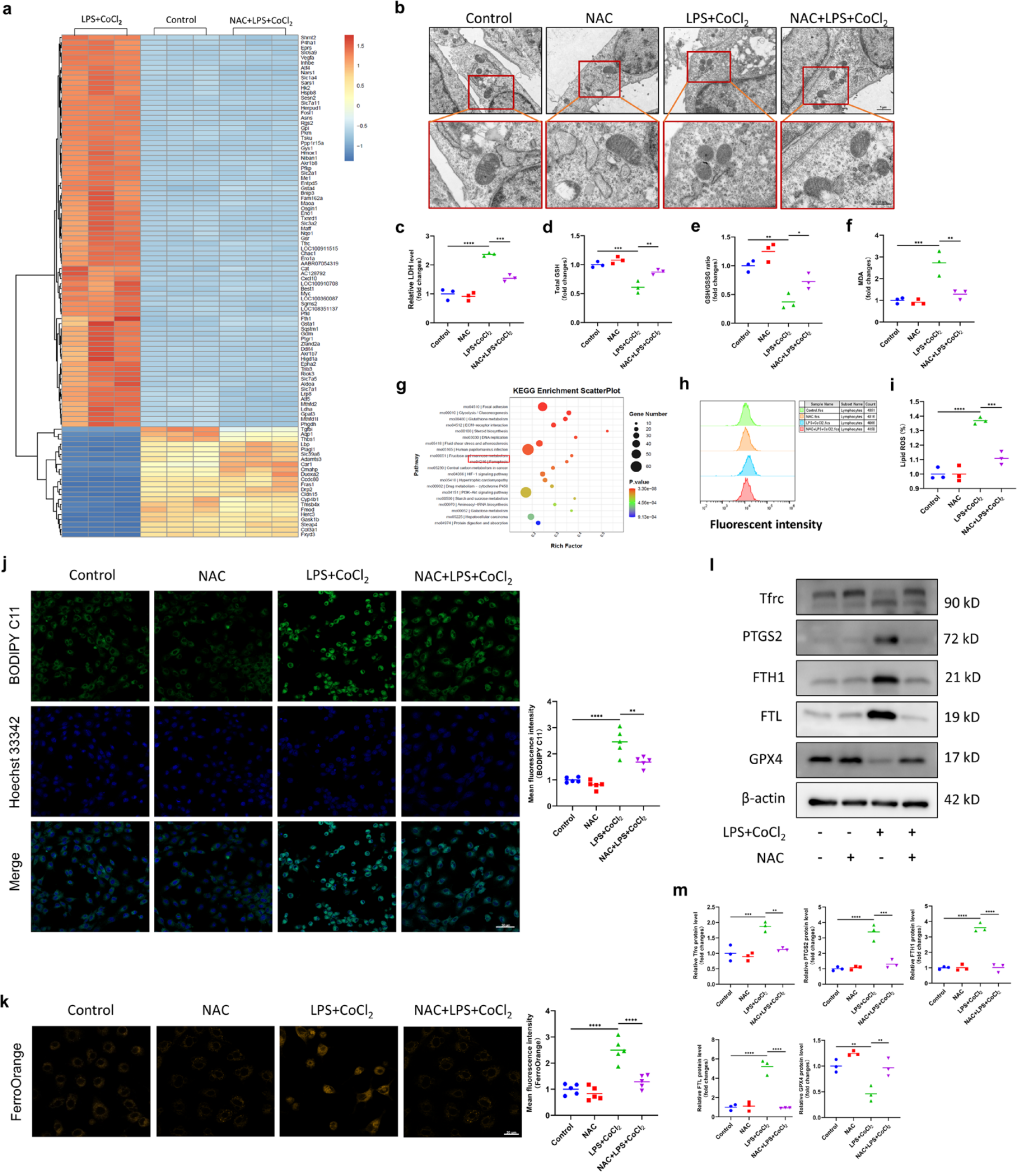
NAC inhibited ferroptosis in IEC-6 cells. **a** Hierarchical clustering analysis and heatmap of the top 100 DEGs. **b** Representative images from the ultrastructure of mitochondria in IEC-6 cells by TEM. Scale bar = 1 µm (upper panel)/ 500 nm (lower panel, enlarged view). **c-f** LDH levels, GSH levels, the GSH/GSSG ratio and MDA content in IEC-6 cells. **g** KEGG pathway enrichment analysis of the top 20 DEGs. **h, i** Lipid ROS levels in different groups detected by flow cytometry. **j** Representative IF images of cell lipid peroxidation detected by BODIPY and quantitative analysis of the mean fluorescence intensity. Scale bar = 50 µm. **k** Representative IF images of intracellular iron content detected by FerroOrange staining and quantitative analysis of the mean fluorescence intensity. Scale bar = 20 µm.** l, m** Western blot analysis of Tfrc, PTGS2, FTH1, FTL and GPX4 expression. ^*^p < 0.05, ^**^p < 0.01, ^***^p < 0.001, ^****^p < 0.0001.

To investigate the underlying mechanism by which NAC exerts its beneficial effects on intestinal cells, we conducted further *in vitro* experiments. We performed RNA-seq analysis of IEC-6 cells in the control group, LPS + CoCl_2_ group and NAC + LPS + CoCl_2_ group. We found that several ferroptosis-related genes, including SLC7A11, Tfrc and FTH1, were differentially expressed among the three groups (Fig. 6a). KEGG analysis revealed that the DEGs were associated with the ferroptosis pathway (Fig. 6g). To confirm that the protective effects of NAC against intestinal cell damage are related to ferroptosis, we further observed the mitochondrial morphology by using TEM. The results showed that there were obvious changes in the mitochondria, including a decrease in mitochondrial volume, an increase in membrane density and a decrease in the number or lack of cristae, in the LPS + CoCl_2_ group. In contrast, mitochondrial morphology in the NAC + LPS + CoCl_2_ group showed milder changes, with a slight increase in the membrane density and clear cristae (Fig. 6b). Moreover, we measured the LDH, MDA, GSH levels and the GSH/GSSG ratio. The results showed that NAC significantly inhibited the increase in LDH and MDA levels while inhibiting the decrease in GSH levels and the GSH/GSSG ratio induced by LPS + CoCl_2_ (Fig. 6c-e). In addition, we evaluated lipid ROS levels by flow cytometry and fluorescence imaging and found that NAC significantly inhibited the increase in lipid ROS levels induced by LPS + CoCl_2_ (Fig. 6h-j)_._ Moreover, intracellular iron overload is another important characteristic of ferroptosis. We measured the intracellular iron content by staining with FerroOrange dye, and the results showed that NAC markedly inhibited the excessive increase in iron content in IEC-6 cells induced by LPS + CoCl_2_ (Fig. 6k). Furthermore, the protein levels of several core factors involved in regulating ferroptosis, including Tfrc, PTGS2, FTH1, FTL and GPX4, were evaluated.

The results indicated that NAC effectively rescued GPX4 expression and suppressed Tfrc, PTGS2, FTH1 and FTL expression following LPS + CoCl_2_ treatment (Fig. 6l, m). These findings suggested that NAC could induce ferroptosis resistance in intestinal epithelial cells.

### SESN2 was Involved in the Effects of NAC on IEC-6 Cell Damage

**Fig. 7:**
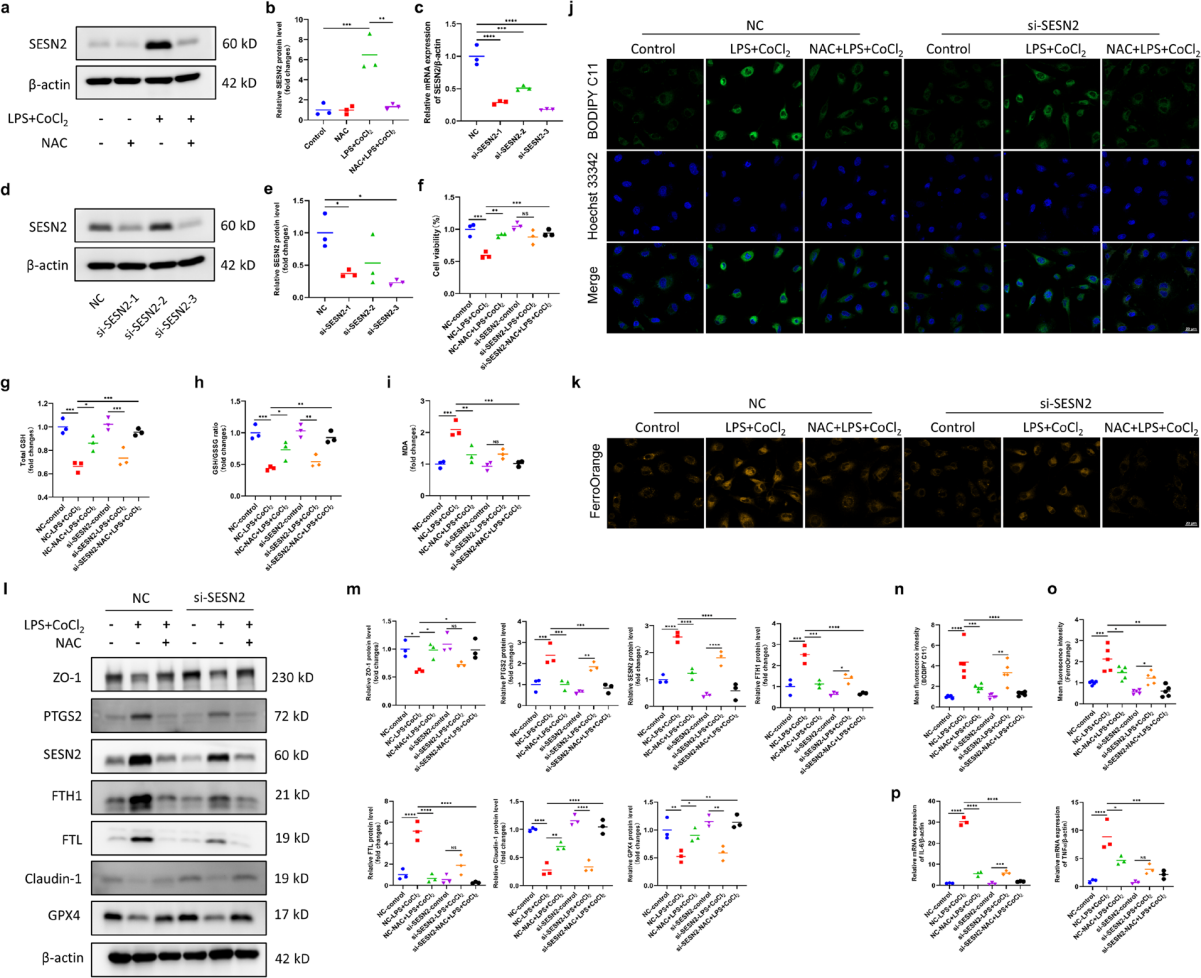
SESN2 knockdown in IEC-6 cells enhanced the positive effects of NAC on ferroptosis, the inflammatory response and barrier damage. **a, b** Western blot analysis of SESN2 expression in IEC-6 cells in the control, NAC, LPS + CoCl_2_ and NAC + LPS + CoCl_2_ groups. **c** qRT‒PCR was utilized to examine the knockdown efficiency of SESN2 siRNA at the mRNA level. **d, e** Western blot analysis was used to examine the knockdown efficiency of SESN2 siRNA at the protein level. **f** Cell viability of IEC-6 cells in the control, LPS + CoCl_2_ and NAC + LPS + CoCl_2_ groups without or with SESN2 downregulation. **g-i** GSH levels, GSH/GSSG ratio and MDA content in each group. **j, n** Representative IF images of cell lipid peroxidation detected by BODIPY staining (scale bar = 20 µm) and quantitative analysis of the mean fluorescence intensity. **k, o** Representative IF images of intracellular iron content detected by FerroOrange staining (scale bar = 20 µm) and quantitative analysis of the mean fluorescence intensity. **l, m** Western blot analysis of ZO-1, PTGS2, SESN2, FTH1, FTL, Claudin-1 and GPX4 expression. **p** mRNA levels of IL-6 and TNFα. ^*^p < 0.05, ^**^p < 0.01, ^***^p < 0.001,.^****^p < 0.0001.

We continued to investigate the mechanism underlying the protective effects of NAC against IEC-6 cell damage. We identified DEGs by RNA-seq analysis and found that SESN2 was differentially expressed (Fig. 6a). We further measured the expression of SESN2 in the different groups by immunoblotting. The results indicated that the expression of SESN2 was significantly increased in the LPS + CoCl_2_ group, whereas the protein level of SESN2 in the NAC + LPS + CoCl_2_ group was significantly decreased (Fig. 7a, b). Hence, we investigated whether the positive effect of NAC on intestinal epithelial cell damage and ferroptosis was dependent on SESN2.

First, we silenced SESN2 in IEC-6 cells via siRNA transfection. Since siRNA-2 was the most effective at suppressing SESN2 expression (Fig. 7c-e), we selected SESN2 siRNA-2 to downregulate the expression of SESN2 in subsequent experiments. The CCK-8 assay showed that in SESN2 knockdown cells, cell death induced by LPS + CoCl_2_ was decreased, and the protective effects of NAC against LPS + CoCl_2_-induced death were increased (Fig. 7f). In addition, the reductions in GSH levels and the GSH/GSSG ratio induced by LPS + CoCl_2_ were inhibited in SESN2 knockdown cells, while the increase in MDA levels induced by LPS + CoCl_2_ was suppressed. Consistently, the protective effects of NAC against LPS + CoCl_2_-mediated damage, as indicated by changes in these indicators, were increased in SESN2-knockdown cells (Fig. 7g-i). Fluorescence imaging showed that the levels of lipid ROS and the intracellular iron content were significantly attenuated in SESN2-knockdown cells following LPS + CoCl_2_ treatment. Consistently, in SESN2-knockdown cells, the ability of NAC to reduce lipid ROS levels and the intracellular iron content was amplified (Fig. 7j, k, n, o). Moreover, after SESN2 was silenced, GPX4 expression increased, and the expression of Tfrc, PTGS2, FTH1 and FTL decreased. Similarly, the ability of NAC to normalize the expression of ferroptosis-related proteins increased (Fig. 7l, m). Furthermore, the levels of barrier proteins and inflammatory cytokines were evaluated to assess cell damage. The results showed the protein levels of both ZO-1 and Claudin-1 were increased and the mRNA levels of both IL-6 and TNFα were decreased in SESN2-knockdown cells. Correspondingly, the ability of NAC to normalize the levels of these indicators increased (Fig. 7l, m, p). Collectively, these findings indicated that a reduced SESN2 level in IEC-6 cells contributed to the inhibitory effect of NAC on ferroptosis, the inflammatory response and barrier damage.

**Fig. 8:**
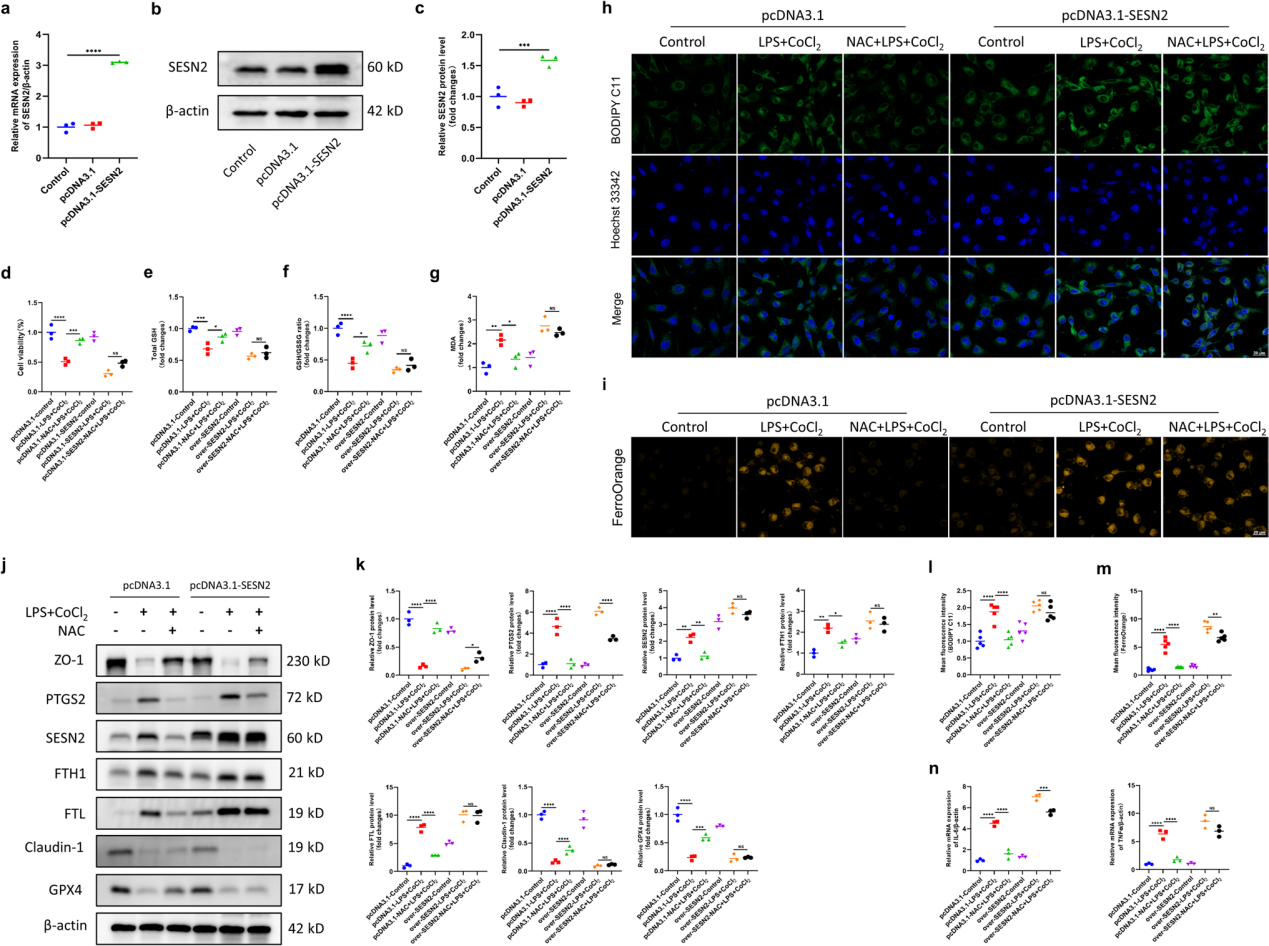
SESN2 overexpression in IEC-6 cells diminished the positive effects of NAC on ferroptosis, the inflammatory response and barrier damage. **a** qRT‒PCR was utilized to examine the efficiency of SESN2 overexpression at the mRNA level. **b, c** Western blot analysis was used to examine the efficiency of SESN2 overexpression at the protein level. **d** Cell viability of IEC-6 cells in the control, LPS + CoCl_2_ and NAC + LPS + CoCl_2_ groups without or with SESN2 upregulation. **e–g** GSH levels, the GSH/GSSG ratio and MDA content in each group. **h, l** Representative IF images of cell lipid peroxidation detected by BODIPY staining (scale bar = 20 µm) and quantitative analysis of the mean fluorescence intensity. **i, m** Representative IF images of intracellular iron content detected by FerroOrange staining (scale bar = 20 µm) and quantitative analysis of the mean fluorescence intensity. **j, k** Western blot analysis of ZO-1, PTGS2, SESN2, FTH1, FTL, Claudin-1 and GPX4 expression. **n** mRNA levels of IL-6 and TNFα. ^*^p < 0.05, ^**^p < 0.01, ^***^p < 0.001, ^****^p < 0.0001.

Next, we examined the effects of SESN2 overexpression on IEC-6 cells. The results showed high SESN2 levels in IEC-6 cells transfected with the SESN2 overexpression plasmid (Fig. 8a-c). The CCK-8 assay showed that the inhibitory effect of NAC on cell death induced by LPS + CoCl_2_ was abolished in SESN2-overexpressing cells compared with that in vector-transfected cells (Fig. 8d). In addition, the protective effects of NAC against decreases in GSH levels, the GSH/GSSG ratio and MDA production were almost completely abolished in SESN2-overexpressing cells (Fig. 8e-g). Fluorescence imaging showed that the ability of NAC to decrease the levels of lipid ROS and the intracellular iron content was nearly abolished in SESN2-overexpressing cells (Fig. 8h, i, l, m). Moreover, the regulatory effects of NAC on Tfrc, PTGS2, FTH1, FTL and GPX4 expression were diminished. Furthermore, we examined the levels of barrier proteins and inflammatory cytokines. The outcomes demonstrated that in SESN2-overexpressing cells, the ability of NAC to normalize ZO-1, Claudin-1, IL-6 and TNFα levels was also diminished (Fig. 8j, k, n).

Taken together, these data revealed that the overexpression of SESN2 in IEC-6 cells abolished the protective effects of NAC against ferroptosis, the inflammatory response and barrier damage.

### NAC Suppressed Ferroptosis by Targeting SESN2

Based on the results of the above series of experiments, it is reasonable to speculate that SESN2 plays a central role in mediating IEC-6 cell damage and regulating the effects of NAC. Subsequently, a molecular docking experiment was performed to predict the binding ability of NAC to SESN2. We found that NAC mainly formed hydrogen bonds with the Trp-344, Trp-394, Tyr-405 and Gln-465 residues of SESN2, affecting the activity of the SESN2 protein (Supplementary Fig. 1a, b).

## DISCUSSION

NEC is one of the most destructive diseases in preterm infants [1, 2, 4, 10]. However, there seems to have been very little progress in developing strategies for the prevention and treatment of NEC over the past several decades [4]. In this work, using both *in vivo* and *in vitro* models, we demonstrated that ferroptosis plays a role in the pathophysiology of NEC and that NAC exerts protective effects against NEC by attenuating the inflammatory response, barrier damage and ferroptosis. SESN2 was found to play an essential role in this process, and an increase in SESN2 expression induced intestinal epithelial cell ferroptosis and diminished the protective effects of NAC.

As NEC results in the death of intestinal epithelial cells [10], studying intestinal epithelial cell death is the basis for elucidating the pathogenesis of NEC. Previous studies have shown that apoptosis is an outcome of NEC, not a cause of NEC, and have suggested that other cell death pathways, such as necroptosis, abnormal autophagy, pyroptosis and ferroptosis, are also involved in this disease [8]. Ferroptosis was discovered as a type of programmed cell death in 2012 [27], and the mechanism and function of ferroptosis have attracted increasing interest. The mechanisms governing ferroptosis are complex and are mainly associated with iron overload and lipid peroxidation [28]. After birth, newborns are commonly exposed to hypoxia, hyperoxia, inflammation, infection, acidosis and other oxidative stresses [29]. Poor antioxidant capacity and iron metabolism cause an increase in ROS and free iron levels, which can lead to ferroptosis through lipid peroxidation. An increasing body of evidence indicates that ferroptosis is involved in a variety of neonatal diseases, including hypoxic-ischemic encephalopathy (IHE), bronchopulmonary dysplasia (BPD) and acute respiratory distress syndrome (ARDS) [30–32]. Dang D et al. recently reported that ferroptosis occurs in NEC and that the expression of ACSL4, which activates the NEC-related Toll-like receptor signaling pathway, is significantly increased in experimental NEC [33]. In addition, they found that ferroptosis might be involved in and be a therapeutic target for transfusion-associated NEC [34]. Herein, we observed changes in the levels of ferroptosis-related proteins and lipid peroxidation in NEC patients. Moreover, intestinal epithelial cell damage *in vitro* was ameliorated by ferroptosis inhibitors. These results elucidate the role of ferroptosis, a previously unrecognized contributor, in NEC, providing an experimental basis for targeting ferroptosis to prevent and treat NEC.

Previous studies have reported that NAC has antioxidant and anti-inflammatory properties that decrease the degree of intestinal damage in experimental NEC [22, 35]. Similarly, in the present study, we verified the effects of NAC in downregulating inflammatory factors and upregulating barrier proteins both *in vivo* and *in vitro*. However, the underlying mechanism needs to be further elucidated. A recent study showed that exogenous supplementation with NAC alleviates 5*α*-dihydrotestosterone- and insulin-induced uterine and placental ferroptosis in polycystic ovary syndrome-like pregnant rats [24]. Another study proved that NAC could decrease ferroptosis in high glucose-treated MDCK cells by upregulating SIRT3 and downregulating SOD2 acetylation and subsequently increasing GPX4 protein levels [25]. Additionally, NAC was found to suppress ferroptosis in cancer cells by inducing the expression of Nrf2, a regulator of cellular free iron responsible for iron-mediated lipid peroxidation [36]. Therefore, we hypothesized that NAC may inhibit ferroptosis in intestinal epithelial cells and further explored the mechanism underlying the effect of NAC in inhibiting NEC-associated ferroptosis. In this study, we reported for the first time that NAC attenuates ferroptosis in NEC by reducing lipid peroxidation and inhibiting iron overload, broadening our understanding of the possible ability of NAC to protect against NEC. However, the regulatory mechanism upstream of ferroptosis needs to be further explored to provide a deeper understanding of how NAC ameliorates NEC.

After confirming the protective effects of NAC against NEC, we further combined the RNA-seq results with reports in the literature to explore the mechanism underlying the effects of NAC on NEC and found that NAC might alleviate NEC by regulating the expression of SESN2. SESN2 is an important member of the sestrin family of proteins and has been widely reported to be a stress-inducible regulator of metabolism [37]. Emerging evidence suggests a connection between SESN2 and ferroptosis. A report illustrated that SESN2 shields dendritic cells from sepsis-induced ferroptosis through the ATF4-CHOP-CHAC1 signaling pathway [38]. In addition, SESN2 was also found to be dependent on Nrf2 and to contribute to protecting against iron overload and ferroptosis-induced liver injury [39]. A bioinformatics-based study revealed ferroptosis-related proteins are differentially expressed in diabetic peripheral neuropathy patients and further confirmed that SESN2 is a key element in the protein‒protein interaction (PPI) network of ferroptosis-related DEGS through PPI network analysis [40]. Because SESN2 has mostly been reported to be a protective factor and is involved in ferroptosis, we speculated that inhibiting the expression of SESN2 might weaken the protective effects of NAC against NEC. However, unexpectedly, this study revealed that SESN2 knockdown alleviated intestinal epithelial cell damage and enhanced the effects of NAC against ferroptosis, the inflammatory response and barrier damage. In contrast, overexpression of SESN2 counteracted the protective effects of NAC on intestinal epithelial cells. These results are in stark contrast to many previous research reports. Consistent with our results, some studies have proven that the upregulation of SESN2 does not exert a protective effect under some conditions. An early study showed that the overexpression of full-length SESN2 cDNA is toxic to many types of cultured cells and leads to cell apoptosis [18]. Another study reported that inactivation of SESN2 partially rescues the emphysema phenotype in COPD model mice [41]. A recent study revealed that the inhibition of SESN2 attenuates cell apoptosis and oxidative stress in an *in vitro* model of diabetic cardiomyopathy [42]. Clinical studies on SESN2 have also reported that it plays a deleterious role in some diseases. For example, Leonidas Angelakis et al. demonstrated that SESN2 is expressed at higher levels in COPD patients with severe emphysema and contributes to the development of COPD [43]. A 36-month follow-up cohort study revealed that SESN2 concentrations in plasma were high in chronic heart failure patients, indicating that the SESN2 increases the incidence of adverse cardiac events [44]. Another study on subjects with diabetes showed that the concentration of SESN2 is increased in patients with metabolic syndrome and is especially related to insulin resistance and a high body fat percentage [45]. These findings differ from the conclusions of most previous studies, suggesting that patients with such diseases might benefit from antagonists of SESN2. Therefore, it seems that the mechanisms underlying the regulatory effect of SESN2 are more complex than expected, and more research is needed to clarify whether SESN2 plays a beneficial or detrimental role in human diseases. Taken together, our findings provide the first clue that SESN2 is a harmful factor in NEC.

At the end of the present study, we predicted the binding mode and affinity between NAC and SESN2 by molecular docking technology, providing a reference and clues for further in-depth exploration of the potential mechanisms by which NAC targets SESN2 to alleviate NEC.

The current study contains several limitations. Due to ethical limitations, the intestinal samples in the control group were not sourced from normal infants; therefore, they may not fully represent true controls. Our current research focused only on intestinal epithelial cells. In future studies, we will investigate the roles of other types of intestinal cells in the pathogenesis of NEC and explore potential therapeutic methods. The findings will be more convincing if ethical consent is obtained to isolate and cultivate immature human intestinal epithelial cells for the establishment of NEC models *in vitro*. In addition, our current research mainly focused on the protective mechanism of NAC in cell models, and further studies using transgenic SESN2 gene knockout or overexpression mice are needed to clarify the role of NAC more thoroughly. Furthermore, we will use biological analysis methods to further explore the specific regulatory mechanisms by which NAC downregulates SESN2 to inhibit ferroptosis and protect against NEC.

## CONCLUSION

**Fig. 9:**
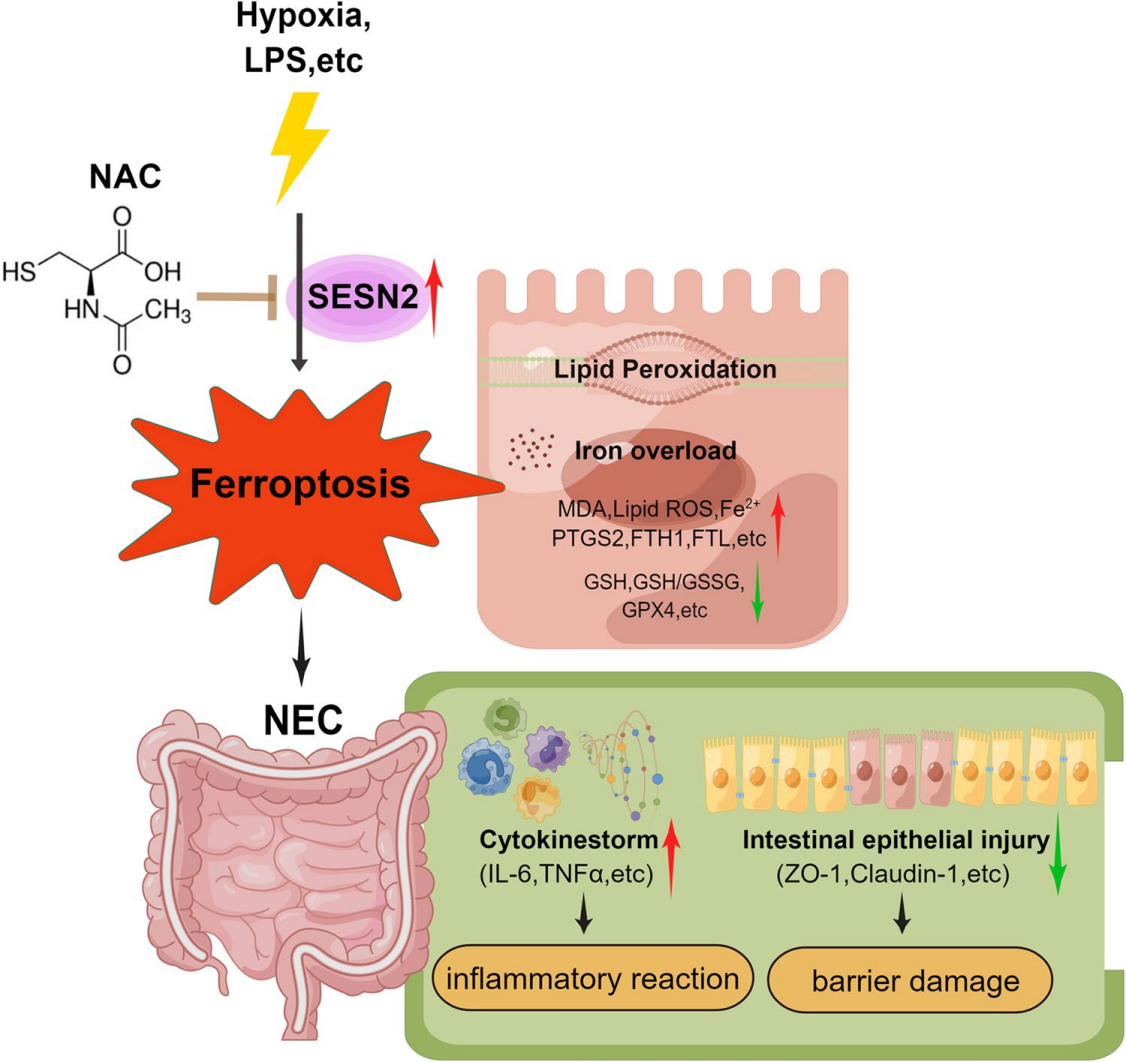
Overview of the potential mechanism underlying the protective effects of NAC against NEC (the figure was created with Figdraw.com).

Our study demonstrated that ferroptosis occurs in NEC and revealed that NAC could attenuate NEC progression by suppressing SESN2 expression to inhibit ferroptosis in intestinal epithelial cells, suggesting that NAC might be an effective clinical treatment for NEC. Therefore, NAC is a potential inhibitor of ferroptosis and is a promising method for preventing and treating NEC in the clinic. Further research is needed to explore the specific regulatory mechanism by which NAC downregulates SESN2 to inhibit ferroptosis and protect against NEC, which may provide new ideas about the underlying mechanism and approaches to NEC interventions, thereby improving the quality of life of newborns, especially premature infants. The potential mechanism for the protective effects of NAC against NEC was summarized in Fig. 9.

## Supplementary Information

Below is the link to the electronic supplementary material.

Supplementary file 1. (PNG 278 KB)

High Resolution Image (TIF 390 KB)

## Data Availability

All supporting the findings of this report are included in this article. The data are available from the corresponding author upon reasonable request.
