# Supplementary figures and images for: N-Acetylcysteine Alleviates Necrotizing Enterocolitis by Depressing SESN2 Expression to Inhibit Ferroptosis in Intestinal Epithelial Cells
Source: Inflammation. 2024 Jul 22;48(1):464–82. doi: 10.1007/s10753-024-02068-5 (PMC11807027; doi:10.1007/s10753-024-02068-5)

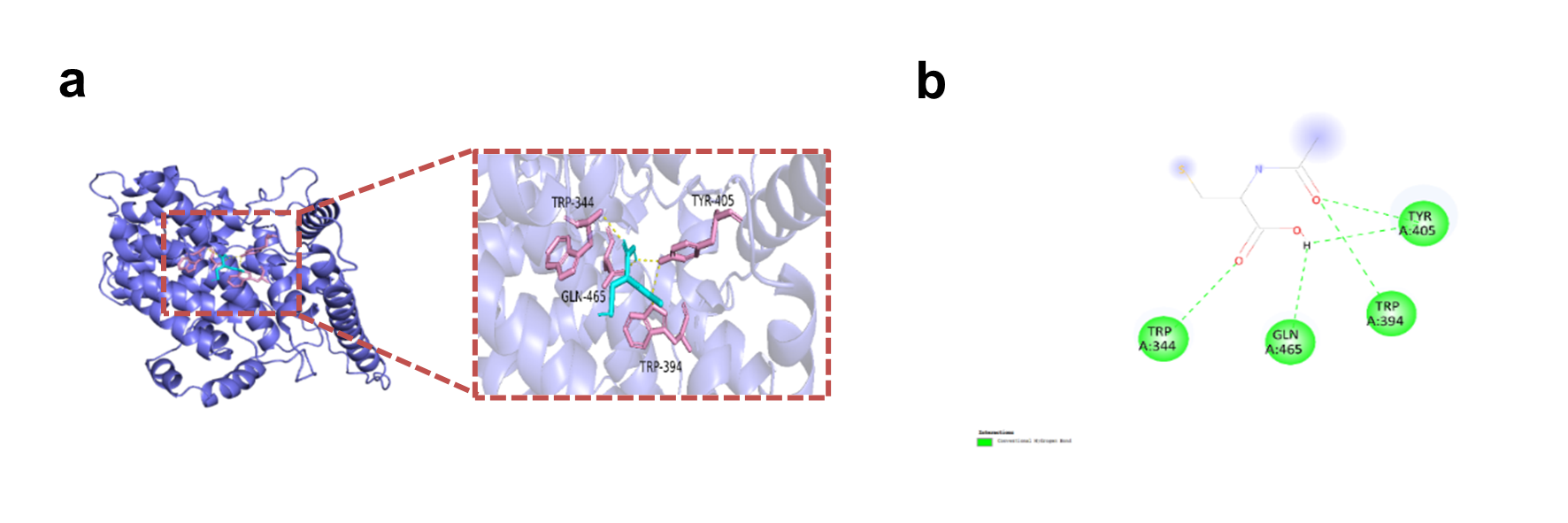

Supplement: Supplementary file 1 — (PNG 278 KB) [file 10753_2024_2068_Fig10_ESM.png]

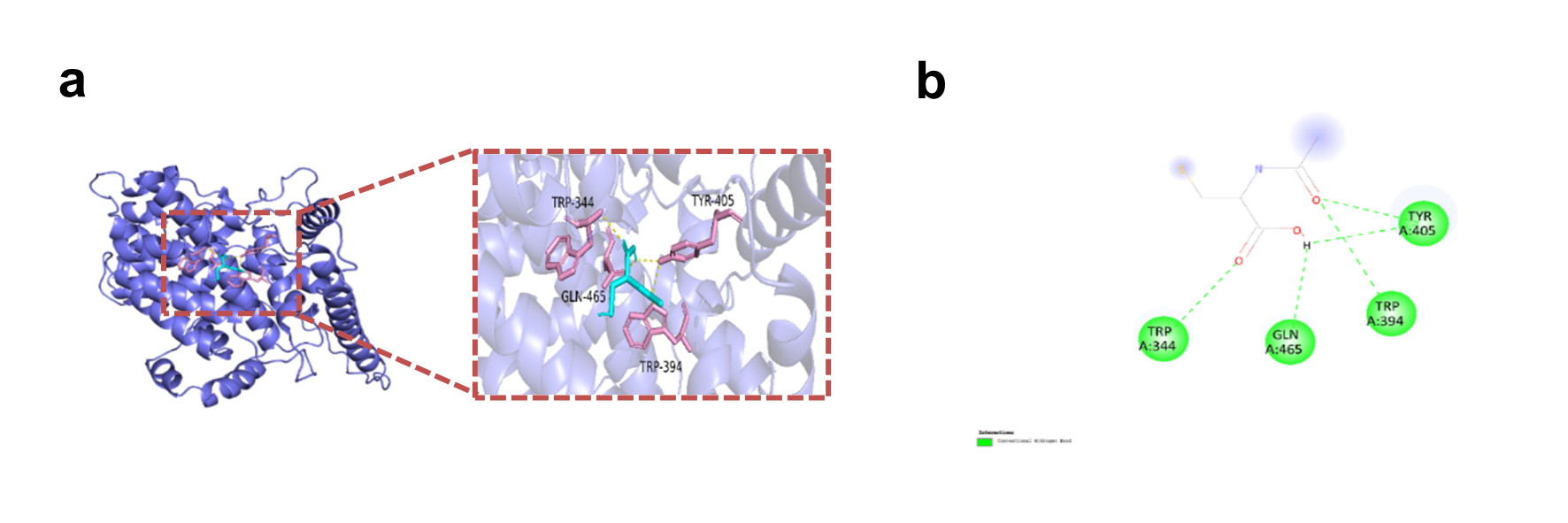

Supplement: Supplementary file 2 — (TIF 390 KB) [file 10753_2024_2068_MOESM1_ESM.tif]
